# The Importance of Including Psychophysiological Methods in Psychotherapy

**DOI:** 10.1007/s10484-024-09667-w

**Published:** 2024-11-02

**Authors:** Paul Lehrer

**Affiliations:** Rutgers Robert Wood Johnson Medical School, Piscataway, NJ USA

**Keywords:** Psychotherapy, Biofeedback, Autogenic training, Progressive muscle relaxation

## Abstract

This paper describes characteristics of sophisticated use of psychophysiological therapy procedures and describes a scoping review of evidence that adding psychophysiological procedures to psychotherapy improves outcome. It also reviews literature describing comparisons between psychophysiological procedures and various CBT and other verbal psychotherapy procedures when used as monotherapies. Some details of progressive muscle relaxation, autogenic training, and biofeedback are described that often are omitted in standard clinical training, including the method of diminishing tensions and differential relaxation training in progressive muscle relaxation, use of autogenic discharges and hypnotic instructions in autogenic training, and resonance frequency training in heart rate variability biofeedback and slow breathing. Although these details are often also missing in outcome studies, tentative conclusions can still be drawn from the empirical literature. As a monotherapy, psychophysiological methods are generally as powerful as verbal psychotherapies, although combining them with psychotherapy yields a larger effect than either approach alone. Psychophysiological methods have their strongest effects on anxiety and depression, with weaker effects for panic and PTSD, particularly when compared with exposure therapy, although the latter comparisons were restricted to relaxation training as a psychophysiological approach. Effects of psychophysiological interventions are weaker among elementary school children than among adults and adolescents. The results suggest that psychophysiological methods should be used along with other psychotherapeutic interventions for greatest effect.

## Introduction

This paper focuses on the role of psychophysiological intervention as a component of psychotherapy. It updates and expands on three previous papers I have written on this topic (Lehrer, 2016, 2018, 2024).

Mental illness is complicated. It is as complex as the human condition itself. Although its many characteristics can be categorized in various ways, this paper focuses on three: cognitive, behavioral, and physiological. Cognitive symptoms include various beliefs or expectations that may present as distorted or false, intrusive, catastrophic, pervasive, persistent, or rigid. Behaviors may be categorized as approach, avoidant, or safety and may be manifest in social and relationship difficulties, inappropriate expression of emotions, immobility, compulsions, eating disorders, substance use, and other problem behaviors. Psychophysiological symptoms include somatic expressions of anxiety, panic, anger, depression, fatigue, appetitive or sleep difficulties, and various somatic sensations and psychosomatic problems.

In our experience, most mental health practitioners are trained in treating cognitive and behavioral aspects of emotional problems, but less training is available for the psychophysiological component. Psychotherapists routinely work with behavior that occurs outside one’s immediate awareness, emotional reactions and how early learning has shaped their expression, the influence of is group norms and communication patterns among family members and associates, characteristics of various important relationships with others, self-image, and irrationality of some pathogenic thought patterns. Some therapists may use such behavioral techniques as behavior rehearsal, social skills training, or operant conditioning. Prominent behavioral components in CBT also include exposure therapy for anxiety and obsessive–compulsive disorders (Exposure Therapy, 2024) and behavioral activation for depression (Behavioral Activation, 2024). But, in the author’s experience the psychophysiological dimension of mental illness is often given short shrift, other than for the use of psychotropic medication and sometimes brief or superficial exposure to progressive muscle relaxation training or, more recently, mindfulness training. Rarely does practice include measurement of psychophysiological events, or many of the subtleties involved in various physiological interventions, detailed understanding of psychophysiological mechanisms by which the methods work, management of side effects, or procedural details that can maximize effectiveness. Although most mental health training includes recognition of some brain mechanisms of behavior and emotion and physiological accompaniments of stress, little emphasis is given to the targeted modification of psychophysiological mental illness components. To compound the problem, as of the writing of this paper, Medicare and many insurance companies do not reimburse for codes related to psychophysiological therapy with psychotherapy for treating psychosomatic disorders (90,876).

The need for treatment specificity by type of problem may have been ignored because cognitive, behavioral, and psychophysiological components of emotional problems often occur together and can exacerbate each other, and improvement in one sphere may sometimes indirectly lead to improvement in others. Indeed, somatic symptoms of stress can be triggered by cognitive distortions (Orzechowska et al., 2021) and relationship problems (Choi & Marks, 2008; Falahatdoost et al., 2020), just as cognitions and coping abilities can be compromised by physical symptoms (Choi & Marks, 2008). Somatic symptoms can be triggered by emotional distress and neurotic thought patterns (Petersen et al., 2024), just as the physical sensations accompanying panic can exaggerate these cognitive and behavioral reactions (Rapgay, 2019). In some cases, the symptoms of somatic diseases and of emotional disorders may overlap, leading to symptom confusion and inappropriate treatment of both disorders. This problem has been well investigated for the comorbidity of asthma and panic disorder, where a number of symptoms are common to both disorders (Feldman et al., 2016; Pateraki et al., 2018), and where symptom confusion often leads to inappropriate treatment for either or both, sometimes with disastrous results (Feldman et al., 2000). Additionally, the presence of somatic symptoms can activate positive feedback loops where perception and sensitivity (i.e., interoceptive sensitivity) to symptoms increases, sometimes aggravating preoccupation with and fear of them, particularly in somatization and panic disorders (Katzer et al., 2012; Nordin et al., 2017; Wolters et al., 2023). The increase in anxiety can then generate a spiraling pattern of increasing symptoms. Perceived lack of control over these symptoms can further exacerbate them and lead to greater disability (Campbell et al., 2018). Psychophysiological interventions that teach people how to alleviate the physical symptoms can lead to a sense of greater control over them, which itself can lead to cognitive and behavioral improvements, including less perceived stress or anxiety, and a willingness to do things that otherwise would have produced symptoms (Kim & Kim, 2018).

Although this paper emphasizes the impact of psychophysiological interventions, similar cross-modality effects also occur for cognitive and behavioral interventions. Verbal psychotherapy can produce psychophysiological changes indirectly, by rendering various thoughts and situations less threatening. It can produce improvements in physical symptoms (Keefe et al., 2019; Lannin et al., 2019). Similarly, behavioral interventions such as exposure therapy for anxiety disorders can produce beneficial cognitive and physiological effects as well as behavioral changes (El-Qirem et al., 2024; Jung et al., 2024; Premkumar et al., 2021). It is perhaps this overlap in effects that prompts many clinicians to target only one of these symptom modalities. This paper examines the question of whether adding a psychophysiological component to therapy can strengthen results.

It is important to note that, although they are often related to each other, the three dimensions of emotional problems (cognitive, behavioral, and physiological) also can sometimes be decoupled. This was demonstrated in series of dramatic laboratory studies by Lang and colleagues several decades ago (Lang, 1968). The investigators instructed snake-phobic people to approach a harmless snake, pick it up, and play with it. They found little correlation between various components of their fear responses: behavioral (how close they came to the snake), cognitive (how much fear they admitted), and physiological reactivity. Would it then be possible that treating only one or two of the symptom modalities might lead to a less effective treatment than if all three were directly targeted? Although various empirical investigations do provide evidence for multidimensional effects of cognitive, behavioral, and physiological domain improvements, this is not always the case, and integrated intervention with components to address each domain may be more effective. This paper specifically addresses the question of whether adding a psychophysiological component to therapy can strengthen treatment effectiveness for psychiatric conditions that inevitably include psychophysiological components as well as cognitive and behavioral ones. Although some of the psychophysiological components may also be affected by psychopharmacological adjuncts to treatment, this large literature will not be included in the current review.

## Some Psychophysiological Systems that are Commonly Dysregulated in Psychopathology

Although allostatic overload from stress and/or disease can affect every system of the body, this section will cover only three physiological systems. These are systems that common psychophysiological interventions directly modify through teaching direct voluntary control: the skeletal muscles, respiration, and heart rate variability.

### Muscle Tension

The skeletal muscles are closely connected to the sympathetic nervous system (Khan et al., 2016; Radovanovic et al., 2015; Rodrigues et al., 2019). Sympathetic arousal governs the fight-fight reflex and is a component of the anxiety condition (Roth et al., 2008). The muscle spindles, cells within the muscles that directly communicate with the central nervous system, are stimulated by sympathetic activity (Jacobson, 1938, pp 231–238) but also are bidirectional, feeding back to the central nervous system (Macefield & Knellwolf, 2018), thereby increasing sympathetic activity when activatedny muscle tension, so that sympathetic arousal both produces muscle tension and is increased by it. Thus, muscle tension is part of a positive feedback loop by which anxiety grows during exposure to threat. My own research has found that instructions to increase muscle tension lead to impairment in the habituation of sympathetically-mediated skin conductance responses elicited by noxious electric shock stimuli (Lehrer, 1972), and Grim (1971) found that induced muscle tension increased anxiety among people who initially had low levels of it. We have found that relaxation training decreases other indices of sympathetic responses elicited by noxious sounds (Lehrer, 1978), and there is a large literature showing that muscle relaxation leads to anxiety reduction, as will be reviewed below.

Muscle tension also is associated with thinking activity. Studies by Jacobson found that muscle tension in the eyes and vocal apparatus increases during active thought and may be a necessary accompaniment to thinking (Jacobson, 1982). He found that mental activity of all kinds is inevitably accompanied by small increases in muscle tension (Jacobson, 1930a, 1930b, 1930c, 1930d, 1931). His work grew from contemporary theory and data on muscular involvement in thought (Dashiell, 1928; Watson, 1924). The finding has been replicated numerous times since then (Boros, 1982; McGuigan, 1966, 1971; Whitham et al., 2008; Zinczenko et al., 1976). Similarly, Max (1937) and McGuigan (1971) found increased sEMG activity in the arms and lips among deaf people. Thus, where anxiety-producing thoughts are part of the positive feedback loop of spiraling anxiety, eliminating facial muscle tension may contribute to blunting the pattern.

Particular patterns of muscle tension tend to be related to emotions and emotionally-laden thought. Jacobson found that patients visiting his clinic had chronically elevated levels of sEMG activity, and, when asked to tense their muscles tightly and then relax, he found that his patients took a longer time than healthy people to return to baseline EMG levels (Jacobson, 1934; Jacobson & Lange, 1959). Elevated muscle tension has repeatedly been found in anxious people (Pluess et al., 2009), particularly in the facial muscles for anxiety (Fridlund et al., 1986; Gomes et al., 2023; Hazlett et al., 1994), depression Teasdale and Bancroft (1977) and PTSD Beck et al. (2006), although several studies have found elevated sEMG levels in anxious people only during or directly after a mild stress situation (e.g., auditory noise) but not during a relaxed state (Balshan, 1962; Goldstein, 1965; Malmo & Shagass, 1949). Notably, muscle tension is mentioned in two of the six DSM-5 criteria for generalized anxiety disorder: muscle tension and restlessness. Muscle tension also is one of the definitional criteria for a panic attack (trembling) and for post-traumatic stress disorder (the startle response). Brain mechanisms for facial muscle tension have recently been found activated among schizophrenic patients during silent thought and hallucinations (Rapin et al., 2012). Increased muscle tension in schizophrenic hallucinations has also been documented (Gould, 1948).

### Breathing

Breathing is regulated by central nervous system processes that usually match the rate and volume of breathing to metabolic needs, such that respiration increases during exercise and decreases during relaxation. The processes controlling respiration involve an interchange among chemical and physical factors in the brain, lung, and blood. Although a neural pacemaker sets average respiratory rhythm (Tryba et al., 2003), the regulatory process also involves periodic sighs that may play a role in both neural control of regulatory rhythm and function of respiratory tissues (Vlemincx et al., 2010, 2013). In addition to responding to metabolic need, respiration can be affected by conditioned reflex pathways by which the body prepares in *anticipation* of respiratory need (Castegnetti et al., 2017). Such processes can both generate and modulate symptoms of stress and anxiety.

Alertness responses, as may become exaggerated in anxiety states, can increase the rate of sighing to the point of hyperventilation (Sargant, 1940). Panic disorder is characterized by a relatively high rate of sighing (Abelson et al., 2001, 2008; Wilhelm et al., 2001), perhaps accounting for the finding of lower Pco_2_ and prominence of hyperventilation symptoms in this group (Blechert et al., 2007; Meuret & Ritz, 2010). Similar findings have been obtained for post-traumatic stress disorder (Blechert et al., 2007), which would be consistent with the presence of dissociative symptoms (Benzakour et al., 2022). In addition to providing more oxygen for an anticipated fight-flight response, a slgh also has a self-soothing component and may help modulate anxiety. A slow deep breath can produce vagal outflow and relaxation during a long exhalation (Van Diest et al., 2014). A sigh can produce a momentary feeling of relief (Vlemincx et al., 2017), as heart rate decreases during a long exhalation. Although this is a parasympathetic (rest and digest) effect, frequent sighs can lead to hyperventilation.

When breathing outpaces metabolic need, a disproportionate blow-off of carbon dioxide leads to alkylosis (increased alkalinity in the blood), which, in turn, causes hemoglobin to develop a greater affinity to oxygen, thus depriving the tissues of oxygen. This reflex is known as the Bohr effect (Bohr Effect, 2024, June 8). When severe, the Bohr effect can cause some of the physical components of anxiety and panic: pounding heart, sensations of smothering, muscle tension and trembling, and mental symptoms of derealization, disassociation, and confusion (Gardner, 1996; Sargant, 1940). Some of these hyperventilation symptoms mimic those of asphyxia and are caused by oxygen deprivation in the heart, muscles, brain, and other organs due to unavailability of oxygen that is stored in hemoglobin. When, additionally, a person fears these body sensations, the anxiety may increase the rate of sighing, in a viscous cycle leading to symptom exacerbation and panic.

### Heart Rate Variability

Heart rate variability reflects and contributes to various reflexes promoting healthy regulation. It consists of several overlapping rhythms, each reflecting a particular regulatory process. One such pattern occurs at respiratory rhythms and is called respiratory sinus arrhythmia (RSA). Heart rate increases during inhalation and decreases during exhalation, thus controlling the amount of oxygen available to the blood (Yasuma & Hayano, 2004). RSA is controlled by the vagus nerve, the parasympathetic nerve regulating visceral function throughout the body, from the gut to the lung. Vagus nerve activity and RSA tend to increase under conditions of relaxation and decrease with stress (Sakakibara et al., 2008). Because the communication between the vagus and the central nervous system is bidirectional (Fawley et al., 2021; Shaffer et al., 2023), changes in RSA can affect parasympathetic activity (the “rest and digest” system), much as parasympathetic activity can increase RSA.

Another regulatory reflex related to HRV is the baroreflex, which modulates blood pressure changes, such that increases in blood pressure cause decreases in heart rate while decreases in blood pressure cause increases, thus modulating changes in blood pressure through rhythmical changes in the amount of blood flowing through the blood vessels (Eckberg & Sleight, 1992). The baroreflex produces a rhythm in heart rate equivalent to the amount of time necessary for blood pressure to change after changes in heart rate, and is largely responsible for the ubiquitous ~10 second Mayer wave in HRV (Ghali & Ghali, 2020). The stress response decreases baroreflex activity, and thus decreases modulation of blood pressure, allowing pressure to rise during anticipation of increased muscular need. However, as is the case with RSA and muscle tension, the baroreflex is bidirectional (Yuan et al., 2019). It is partly controlled by the vagus nerve and changes in baroreflex activity via the vagus nerve can mediate changes in general emotional arousal (Silvani et al., 2015). Baroreflex strength also reflects both the amplitude and complexity of the body’s regulatory systems. As such, it is related youth (Studinger et al., 2009), aerobic fitness (Heydari et al., 2013), and viability among patients with life-threatening illness (Fang et al., 2020; Huang et al., 2024; Thayer & Lane, 2007), and is decreased by various diseases and disorders (Kaufmann et al., 2020). Decreased heart rate variability is a predictor of mortality from a number of causes (Lin et al., 2024; Pradhapan et al., 2014; Yanagisawa et al., 2024; Zeid et al., 2023).

A slower rhythm, approximately 2–3 times per minute, controlled by the alpha sympathetic system, reflects the vascular tone component of the baroreflex, through which blood vessels dilate in response to increases in blood pressure and constrict in response to decreases (Shaffer et al., 2023; Vaschillo et al., 2011; H. Wang et al., 2024). Although less widely studied than the heart rate component of the baroreflex, the vascular tone component also probably plays a role in total body regulation. Other regulatory rhythms, both slower and faster, may also impact heart rate variability. Thus, the amplitude and *complexity* of heart rate variability reflect the adequacy of the body's regulatory systems. Decreased baroreflex activity may affect various bodily symptoms and also may directly affect emotional regulation through central autonomic nervous system interactions (Thayer & Lane, 2000; Thayer et al., 2012).

Usually all three rhythms, those produced by the RSA and the heart rate and vascular baroreflexes (and perhaps more rhythms), are represented in normal heart rate variability. Since all reflect an aspect of homeostatic control, it is not surprising to note that health, fitness, and general resilience all are represented both by amplitude and complexity of heart rate variability (i.e., representing both the strength and quantity of operating control reflexes) (Crameri et al., 2020; Six Dijkstra et al., 2023; McCraty & Shaffer, 2015; Wu et al., 2009). Heart rate variability tends to decrease with age in adulthood (Aschbacher et al., 2024; Shaffer & Ginsberg, 2017), and is related to aerobic fitness (Manresa-Rocamora, 2021; Schimpchen et al., 2023). It decreases in depression (Koch et al., 2019), stress (Kim et al., 2018), anxiety (Chalmers et al., 2014), as well as many other emotional (Jung et al., 2019) and physical disorders (Benichou et al., 2018; Forte et al., 2022; Kubota et al., 2017; Zhao et al., 2024).

## Psychophysiological Treatment Methods and Some Technique Subtleties

There are various circumstances under which a therapist might find it useful to employ a psychophysiological intervention as a psychotherapy component. Most obviously it includes conditions where psychophysiological symptoms are prominent complaints, such as in somatization disorders, somatic disorders aggravated by stress, and panic disorder. Also, as mentioned previously, somatic symptoms are prominent components of many other psychological problems including most anxiety and mood disorders.

Another criterion for the use of psychophysiological interventions is the client’s motivation to learn how to control physiological symptoms and credibility of the treatment in the eyes of the client. This can be particularly important for the client who thinks that their problems are mostly physical (Alfonsson et al., 2016; Epstein et al., 2012) or feels less mental health stigma associated with biofeedback or relaxation than with psychotherapy. Also, various cultures explain mental symptoms in psychophysiological terms (e.g., the Chinese beliefs in energy flows or Indian beliefs in chakras) thus generating particular comfort with a psychophysiological approach. Additionally, some people have been trained to recognize the effects of stress on performance and physical methods to control it, but may need more detailed or targeted help. Most athletes, dancers, actors, and musicians receive such training. In his early research with electrophysiological assessment of muscle tension, Jacobson (1936) found faster than average EMG recovery times among members of a university football team than in the general public, but not as fast as among individuals trained in progressive muscle relaxation. Similarly, singers and players of wind instruments often have received training in relaxed abdominal breathing, but nevertheless benefit from training with heart rate variability biofeedback (Thurber et al., 2010).

Success of a therapy also is often related to the confidence a patient has in it (Ponten et al., 2024). People differ in preferences for various methods and in the methods they find most helpful (Hyland et al., 2016; Lehrer & Woolfolk, 2021). This may not be just a suggestion or placebo effect. Belief in the potency of an intervention and preference for a particular form of treatment are related to adherence to a variety of psychological and medical treatments (Gasslander et al., 2021; Horne et al., 2013). For psychophysiological therapies, this can translate to time spent practicing various techniques and willingness to use them to manage symptoms. Involvement in any psychophysiological treatment program involves some discipline and an ability to concentrate on the method.

Some personality characteristics also predict usefulness of particular kinds of treatment. For example, people with high hypnotic susceptibility tend to take autogenic training more seriously, practice it more, and have better results with it (Spinhoven & ter Kuile, 2000). In my clinical experience, I have found that some people with training in a muscular skill (e.g., athletes, instrumental musicians, etc.) gravitate more to progressive muscle relaxation, while some people influenced by Asian culture gravitate to meditation methods, some trained in breathing (e.g., singers and players of wind instruments) gravitate to breathing methods, and some with a scientific or technological bent gravitate to biofeedback. Patients with somatization disorder often gravitate toward psychophysiological treatments when they are convinced that their symptoms entirely result from a poorly diagnosed somatic disease and a physiological explanation (e.g., hyperventilation effects) can be given. They are particularly sensitive to stigma related to mental illness (Gevirtz, 2021) and often come to psychotherapy with skepticism, as a last resort after failure of various medical interventions that may produce iatrogenic effects (Woolfolk & Allen, 2006).

Here I will describe three psychophysiological methods with a particularly large amount of empirical support and will emphasize some subtleties in each of them that require specialized therapist knowledge and training. Understanding the physiological and psychological underpinnings of each method also is important because effectively communicating the rationale can help foster adherence. In my experience, the formal training required to explain and deliver these therapies effectively is often lacking in psychotherapy training programs. Combined use of psychophysiological methods with psychotherapeutic modalities that target cognitive or behavioral symptoms will also be discussed, even where psychophysiological interventions may involve only a minor proportion of psychotherapeutic time. As part of the treatment package, training to control various psychophysiological symptoms improves the perceived ability to control them and can lead to a greater sense of self-efficacy and general emotional improvement, as found in diverse populations (Asselmann et al., 2023; Lim et al., 2014; Paul & Garg, 2012).

### Progressive Muscle Relaxation

Many psychotherapists have at least a passing acquaintance with progressive muscle relaxation. Edmund Jacobson (1938) designed this method early in the last century. Although he primarily used it to treat psychosomatic problems, he also applied it to emotional problems, particularly anxiety.

Progressive muscle relaxation directly addresses elevated muscle tension with targeted intensive instruction on muscles contributing to anxious thoughts as well as emotions (Jacobson, 1964, 1967). Among individuals not trained to relax, measurable muscle tension tends to remain even when they think they are relaxing (Jacobson, 1942), but it decreases markedly after training (Jacobson, 1934; Lehrer et al., 1997).

In trained individuals, sensitization to muscle tension can also facilitate decreases in cognitive, emotional, and somatic symptoms. Reviews of empirical literature have found that practice of progressive muscle relaxation decreases anxiety (Conrad & Roth, 2007; Francesco et al., 2010; Manzoni et al., 2008), depression (Y. Wang et al., 2024), fatigue (Y. Wang et al., 2024), and symptoms of various stress related physical disorders (Carlson & Hoyle, 1993; Jacobson, 1938). In addition to treating various aches and pains, the method is an effective adjunct (and sometimes principal) treatment for anxiety, insomnia, hypertension, and functional gastrointestinal symptoms (Jacobson, 1938, chapter 19). In his early work, Jacobson published radiological studies of the gut among people with irritable bowel syndrome (Jacobson, 1927), showing how the diameter markedly increased along with decreases in symptoms of pain, constipation, and diarrhea.Although findings of clinical improvement are sometimes stronger than those of decreased muscle tension as measured by the surface electromyogram (Conrad & Roth, 2007), it is possible that muscle findings are influenced by conditions of testing, because muscle tension can vary widely within individuals depending on task demands and the site of muscle measurement, and because tension usually is not measured in many sites where tension may remain. Also, clinical improvement can be influenced by concentrating on muscles rather than various worries, and by belief in the potency of the training.

The progressive muscle relaxation method in common practice has appreciably changed from Jacobson’s original method. There are theoretical reasons to think that some of these changes may have decreased effectiveness. For example, people are now often taught to tense various muscle groups tightly, and then relax, under the assumption that a “rebound effect” will cause greater muscle relaxation afterward (Bernstein & Borkovec, 1973). Although widely accepted, this theory has been disproven, because large muscle contractions tend to produce elevated residual tension after tension release (Lehrer et al., 1988) and this may decrease sensitivity to more subtle levels of tension. This is a corollary of Weber’s law for perception of change and Stevens’ psychophysical power function. Weber’s law states that larger baseline levels of a stimulus increase perceptual threshold for perceiving differences in stimulus intensity. The law was amended by Fechner to apply a logarithmic function to the relationship between baseline intensity and threshold for perceiving differences (Weber-Fechner law, 2024). Informed by this, Stevens’s found a logarithmic relationship between subjective numerical ratings of stimulus intensity and actual intensity, with greater differentiation at lower levels of stimulation (Laming, 1997). Although we have found no studies of these functions for interoception of muscle sensations (or any form of interoception), they have been applied to perception of light, heat, pain, etc., and, since they appear to be universal, they probably apply to perception of muscle sensations as well, so greater sensitivity to low levels of muscle tension might be expected following minimal muscle contractions. No studies have yet been done to validate this theory.

In my experience as a trainee of his, Jacobson used the “method of diminishing tensions” to decrease perceptual threshold of muscle tension. In this procedure the individual tenses each muscle progressively smaller amounts until tension is almost imperceptible to the observer (Jacobson, 1938, pp 185–186). Eventually, muscle sensations can be perceived even when the person only *thinks* of tensing a muscle, and to the point where the individual becomes sensitive to the small sensations associated with underlying muscle tone (Jacobson, 1938, chapter 13). Then the instruction is given to “go negative,” or relax completely, to the point of zero muscle tension. Deliberate tension is used not to induce relaxation but as a didactic procedure for learning to recognize and control small amounts of tension, the kind that is often present in everyday life, and learning to eliminate it. In his research and clinical work Jacobson (1938) measured muscle tension first by palpation of various muscles, and eventually by using surface electromyography (sEMG), a method he developed collaborating with engineers from Bell Laboratories. Surface EMG biofeedback now is a commonly used biofeedback method for relieving various tension symptoms, including headache, backache, and temporomandibular joint pain as well as for treating fecal or urinary incontinence (Criswell, 2011). Jacobson advised against using sEMG biofeedback rather than progressive muscle relaxation and the method of diminishing tensions because he did not want his patients to rely on a machine to tell them when slight amounts of muscle tension remained. Jacobson also eschewed the use of hypnotic-like suggestion, which is a common accompaniment of more recent adaptations of progressive muscle relaxation methods. Jacobson’s rationale was that there could be a difference between *thinking* that a muscle is relaxed vs. actually *being* relaxed, with zero muscle tension as measured by electromyography often as the criterion (Jacobson, 1938, chapter 15). Although Jacobson’s early descriptions of the method indicated that it involved many sessions over many months, this is not a necessary characteristic of his method and was not, in fact, the way he often worked. He has described cases where training was quite brief (Jacobson, 1938 chapter 18; 1964). In addition to treating various aches and pains, the method is an effective adjunct (and sometimes principal) treatment for anxiety, insomnia, hypertension, and functional gastrointestinal symptoms (Jacobson, 1938, chapter 19). In his early work, Jacobson published radiological studies of the gut among people with irritable bowel syndrome (Jacobson, 1927), showing how the diameter markedly increased along with decreases in symptoms of pain, constipation, and diarrhea.

In Jacobson’s method, special training is given to the facial muscles, particularly those around the eyes, mouth, and speech muscles for patients with obsessive thoughts and insomnia, because of involvement of these muscles in the process of thinking. He found that relaxing these muscles decreases all kinds of mental activity. (Jacobson, 1938 chapter 10). Progressive muscle relaxation including these areas of the body also has been applied to treating hallucinations in schizophrenia (Slade, 1973). Jacobson also found that progressive muscle relaxation training decreased the startle reflex, one of the common symptoms of post-traumatic stress disorder (Jacobson, 1938).

Another aspect of Jacobson’s method that is more often incorporated in contemporary psychotherapy is his use of “differential relaxation” training (Jacobson, 1943), sometimes called “applied relaxation” (Ost, 1987). This involves training patients to use muscle relaxation when they are in situations that ordinarily would evoke tension-related symptoms. Jacobson specifically taught his patients how to relax while doing other activities such as reading or conversing (Jacobson, 1938, chapter 6). The patient is then taught to apply differential relaxation skills when symptoms of stress occur.

The mechanism by which progressive muscle relaxation might decrease anxiety and emotional activation was outlined by Gellhorn (1958), who performed experiments showing that chemically induced peripheral muscle relaxation through curare produced somnolence in animals. Curare blocks transmission at the neuro-muscular junction causing complete muscle flaccidity. Gellhorn considered this as evidence for the mechanism by which progressive muscle relaxation works. More recent evidence has borne out his interpretation. We have found evidence of decreases in peripheral measures of sympathetic arousal and increased parasympathetic activity produced during Jacobson’s method of progressive muscle relaxation (Lehrer, 1978; Lehrer et al., 1997).

### Autogenic Training

Autogenic training, a self-hypnotic technique, was developed in Germany by Johannes Schultz at about the same time as Jacobson was developing progressive muscle relaxation in the United States. It is a self-hypnotic method that focuses on changes in perception of muscle tension and blood flow in various parts of the body. There is evidence that some changes in muscle tension and blood flow do take place through hypnotic suggestion (Luthe, 1970). Training begins with six standard exercises, mostly related to relaxation experience, decreased sympathetic arousal, and increased parasympathetic tone: perception of heaviness and warmth in the limbs, calm heartbeat, cool forehead, automatic breathing, and warmth in the gastrointestinal area (Linden, 2021; Schultz & Luthe, 1969, chapter 4). It contrasts with Jacobson’s method in that it uses mental activity rather than direct physiological training to control various body organs and makes specific use of hypnotic suggestion; but the emphasis still is on the psychophysiological. In addition to the six standard exercises, full use of the autogenic method may involve suggestions to change blood flow and relax muscles in other parts of the body, as well as suggestions for a calm mind, habit change, and reduction of unwanted thoughts (Schultz & Luthe, 1969, chapter 6). A meta analysis by Stetter and Kupper (2002) found medium to high effect sizes for treating a variety of problems, with largest effects on various mood, cognitive performance, quality of life, and physiological variables, with consistent effects across multiple studies for tension headache/migraine, mild-to-moderate essential hypertension, coronary heart disease, asthma, somatoform pain disorder, Raynaud's disease, anxiety disorders, mild-to-moderate depression/dysthymia, and functional sleep disorders. A more recent meta-analysis (Kohlert et al., 2022) found consistently positive effects for various pain disorders.

In our clinical experience the method is particularly effective among people with higher levels of hypnotic susceptibility. It tends to be disliked among people who score low on the Stanford Clinical Scale of Hypnotic Susceptibility (Spinhoven & ter Kuile, 2000). Nevertheless, perhaps because the six standard exercises are most like the easiest tasks on tests of hypnotic susceptibility, we have found some benefit among people with moderate or even low levels of hypnotic susceptibility.

An important aspect of the autogenic technique that is well known to hypnotherapists but often omitted in training manuals is a focus on “nonintentionality.” During training, the trainees are specifically instructed not to *try* to achieve any particular bodily sensation or mental activity. They are asked just to *imagine* what a sensation might be like, and to observe passively what happens. This instruction is like the procedure described by the hypnotherapist Milton Erickson as “pacing and leading,” whereby the patient is instructed just to do and feel what they currently are doing and feeling while undergoing a hypnotic induction that gradually gives specific instructions to produce therapeutic effects (Hollborn, 2022; Jacquin Hypnosis Academy, 2023).

Another aspect of autogenic training that often is omitted from current manuals is the use of what Schultz called “autogenic discharges” (Luthe, 1973). These come in the form of sudden increases in pain or other body discomfort, or sudden increases in anxiety, depression, or anger, sometimes accompanied by reawakening of long-forgotten memories. Using the imagery of the steam turbine, a common model for understanding emotion in the early 20th century, Schultz described these events similarly to letting off steam.He called them “abreactions.” He noted that major symptoms often improved immediately after such experiences, including headaches, anxiety, and depression (Schultz & Luthe, 1969 chapter 7). In my own practice I have found these to be rare occurrences. Still the reawakened memories occasionally become productive for psychotherapy, leading to the resolution of some important problems. A school of psychoanalysis in Europe has found an overlap in mechanisms for improvement in psychoanalysis and autogenic training. They combine both methods in treatment (Gonzalez de Rivera, 1997; Vespa & Sirolla, 1996), and sometimes use autogenic training to access unconscious memories and feelings. Although there have been no studies of autogenic training’s effect on the brain, these “discharges” may represent a form of emotional disinhibition (Huey, 2020) like that occurring in dreams, where emotional experiences are often exaggerated from those occurring during awakeness. It is known that prefrontal cortical activity tends to actively inhibit emotional events (Kenwood et al., 2022). This area of the brain becomes less active during dreams (Northoff et al., 2023). There is evidence that the emotional content in dreams may have adaptive functions in control of emotion (Samson et al., 2023). Perhaps the same mechanism explains some autogenic training effects. Autogenic training has been used effectively for treating various anxiety and mood disorders (Luthe &

Schultz, 1966).

### Heart Rate Variability Biofeedback

Much has been written in this journal about heart rate variability biofeedback (HRVB). This procedure teaches people to breathe at a slow rate that maximizes the amplitude of heart rate changes co-occurring with each breath (i.e., resonance breathing, see below). A meta-analysis found that HRVB produces medium to large effects on reducing anxiety, depression, anger, and systolic blood pressure, while also improving athletic and cognitive performance (Lehrer et al., 2020). As with muscle tension and progressive muscle relaxation, clinical improvements do not universally correlate with changes in baseline heart rate variability. Thus, although heart rate changes with breathing are controlled through the parasympathetic system, clinical improvement is thus not always related to increases in parasympathetic tone. However, immediate increases in HRV during training are huge. We have interpreted the salutary effects as stemming from exercise of homeostatic reflexes during HRVB practice sessions, thereby strengthening them (Lehrer et al., 2003).

Although the average rate of breathing to produce this effect is 5.5 times per minute (about 11 seconds per breath) (Vaschillo et al., 2006a, 2006b, 2006c), the rate of maximum amplitude varies among people, usually in the range of 4.5–6.5 breaths per minute, and corresponds to a resonance frequency inherent in the baroreflex system (Vaschillo et al., 2002, 2006a, 2006b, 2006c). When breathing at this rate, respiration stimulates and strengthens the baroreflex (Lehrer et al., 2003; Vaschillo et al., 2002), a reflex that regulates blood pressure. The baroreflex is controlled through nucleus tractus solitarius (Rogers et al., 2000), the brain center controlling homeostatic activity for many autonomic functions affecting the cardiovascular, respiratory, and gastrointestinal systems (Andresen & Paton, 2011). Probably because of these connections, strengthening of the baroreflex by exercising it through resonance frequency breathing produces improved general regulation and beneficial effects on disorders affecting multiple organs.

The baroreflex causes a well-known rhythm in heart rate at about 0.1 Hz or six times a minute, sometimes called the “Mayer wave.” When people engage in resonance frequency breathing, respiratory sinus arrhythmia interacts with the baroreflex, such that respiratory-induced increases and decreases in heart rate coincide with baroreflex-induced increases and decreases. The results produce an oscillation in heart rate greater than the sum of these two reflexes, a characteristic of resonance (Vaschillo et al., 2006a, 2006b, 2006c). The more closely respiration rate is to the resonance frequency of the system, which is specific for each individual, the larger the amplitude of heart rate variability that this produces at the respiratory frequency (Vaschillo et al., 2002). With resonance thus being the mechanisms of HRVB, identifying a personalized breathing rate that stimulates it can easily be determined by breathing at various frequencies within this range and finding the frequency that produces the largest-amplitude oscillations in heart rate. There is evidence that breathing more closely to resonance frequency produces larger clinical effects on anxiety than breathing slightly faster (Steffen et al., 2017). A similar relationship was found for blood pressure control (Chen et al., 2016).

Recent fMRI research has found that regular practice of HRVB increases connectivity between limbic structures that generate emotion and cortical structures that modulate it in the ventromedial prefrontal cortex (VMPFC) (Nashiro et al., 2023). Higher heart rate variability is associated with greater VMPFC activity (Maier & Hare, 2017), and changes in anxiety symptoms are associated with changes in connectivity between the amygdala and the VMPFC (Makovac et al., 2016).

## Scoping Review of Literature on Psychophysiological Treatments

In this review, I looked at effects of adding psychophysiological treatments to psychotherapy, the comparative effects of psychophysiological treatments and verbal psychotherapy, and adding psychotherapy to psychophysiological treatments. I searched Ovid PsycInfo and Medline for papers, using the following syntax: (biofeedback or relaxation or autogenic) and (psychotherapy or cognitive therapy or cognitive behavior therapy or CBT or psychoanal*).ti. This search yielded 116 papers. In this review 25 studies isolated the comparisons of interest. Results of seven meta-analyses also are described.

### Adding Psychophysiological Treatments to Psychotherapy

Since most psychological treatment involves some form of verbal psychotherapy and since there is a large body of literature attesting to effectiveness of exposure therapies, it is particularly important to examine the effects of adding a psychophysiological component to other forms of ongoing therapy, to see if there is added benefit.

The review found that adding a psychophysiological therapy to verbal or behavioral therapies produces stronger therapeutic effects for anxiety and depression. Shiga et al. (1999) found that adding autogenic training to cognitive behavior therapy produces greater effects in reducing symptoms of social phobia. Krampen (1999) found that adding autogenic training to psychotherapy has greater effects than psychotherapy alone for treating depression. Buhler (2017) studied patients with mixed anxiety and depression diagnoses and found that adding progressive muscle relaxation training to an ongoing program of psychoanalytically oriented psychotherapy produces greater decreases in both conditions. Caldwell and Steffen (2018) found that adding HRVB to psychotherapy produces greater decreases in depression and increases in heart rate variability than psychotherapy alone. Krampen (2015) found a larger long-term reduction in depression where either progressive muscle relaxation or autogenic training is added to psychotherapy than when psychotherapy is given alone. However, some psychophysiological therapies may not strengthen effects of exposure therapy for panic disorder. de Ruiter et al. (1989) found that adding breathing retraining to exposure therapy for panic disorder is not more effective than exposure therapy alone. Michelson et al. (1996) added either relaxation or cognitive therapy to graded exposure for treating panic disorder with agoraphobia and found significantly greater effect only for adding cognitive therapy. Nevertheless, because exposure therapy can be aversive, there is evidence from other sources that there may be a greater willingness for people to engage in exposure after first having learned to control the physiological components of their anxiety reactions (Meuret et al., 2012). However, in a cautionary note, Furukawa et al. (2021) found in a meta-analysis that relaxation therapy may be harmful for treating depression, whereas behavioral activation is helpful.

The results are less consistent for children. Eisen and Silverman (1993) found no additional benefit from adding relaxation therapy to cognitive therapy for treating anxiety among overanxious children. Caldwell et al. (2019) performed a meta-analysis on studies of school-based CBT programs (most including relaxation therapy) to prevent or reduce anxiety and depression in primary and secondary school students. They found that relaxation-based interventions are effective in reducing anxiety among secondary school students, but there they found little evidence that CBT reduces depression in either secondary or primary school students, or that anxiety is reduced or prevented by either method among primary school students.

### Comparison Between Effects of Psychophysiological Therapy and Verbal Psychotherapy

Some studies found larger effects for psychophysiological therapies than for verbal or behavioral therapies, although some show the contrary, leading us to conclude that all of these approaches have similar effects when used as monotherapies, with the possible exception of biofeedback to increase end-tidal carbon dioxide, which has a particularly large effect size in treating panic (Lehrer et al., 2020; Leyro et al., 2021). Our review of respiratory therapies for treating anxiety (Leyro et al., 2021) showed that treatments with a respiratory component have a medium to high effect size for reducing anxiety, and that respiratory interventions with a biofeedback component have a significantly higher effect size than those without this component. Arntz (2003) found overall equivalent long-term effects for applied relaxation and cognitive therapy for treating generalized anxiety disorder, but with larger short-term effects for the relaxation condition. In a meta-analysis of late-life anxiety, Hafeez and Holsinger (2022) found that both relaxation therapy and cognitive therapy are more effective than no treatment, but so are various other active treatments, all of which are more effective than no treatment in reducing anxiety. Relaxation therapy was found to have greater effects than cognitive therapy. In a meta-analysis, Yusufov et al. (2019) evaluated various stress management methods among undergraduate and graduate students. They found that relaxation therapy has greater stress reduction effects than CBT, coping skills training, mindfulness training, and psychoeducation. Lo et al. (2018) conducted a meta-analysis of stress management strategies among students in health-related professions. They found a larger effect size on anxiety for relaxation therapy than CBT for anxiety and depression. Achmon et al. (1989) found greater decreases in blood pressure among hypertensive individuals receiving biofeedback to decrease heart rate during stressful situtions than for cognitive anger control therapy, but both conditions produced greater blood pressure changes than a control condition. Bell et al. (1983) found greater effectiveness for biofeedback therapies than for eclectic psychotherapy for eliminating psychological symptoms among people with headaches. A meta-analysis for depression among adults by Li et al. (2020) found relaxation therapies to be helpful for depression compared with a combination of waiting list and active psychotherapeutic comparison groups.

Other studies found larger effects for cognitive or behavioral interventions than for psychophysiological ones, particularly where the psychophysiological therapy primarily consisted of an abbreviated form of progressive relaxation. This psychophysiological interventions tend to be particularly weaker among children for anxiety disorders. Bilek et al. (2022) found larger effects for exposure-based CBT then a relaxation-focused treatment including a simple form of progressive muscle relaxation among children and adolescents. In a meta-analysis of CBT studies for childhood anxiety, Clark et al. (1994) compared applied relaxation therapy, cognitive therapy, and imipramine treatment among patients with panic disorder. Although all three treatments were effective in reducing panic symptoms, cognitive therapy was found to be more effective than both applied relaxation therapy and imipramine at three-, six-, and 15-month follow-up periods.

Similar findings have occurred among adults. Whiteside et al. (2020) found that interventions including relaxation training in the CBT practice are significantly less effective in reducing anxiety than interventions that omitted it and give greater emphasis to cognitive restructuring and exposure. All interventions, however, were found to be effective with a medium to large effect size. Hinton et al. (2011) found larger effects for CBT than for a method of applied relaxation training for treating patients with treatment-resistant PTSD. Yusufov et al. (2019) found greater effects for CBT than relaxation therapy for treating symptoms of perceived stress. Lo et al. (2018) meta-analysis found greater effects for mindfulness or CBT than for relaxation training in treating perceived stress. Bogels (2006) found greater long-term effects of training in task concentration combined with cognitive therapy than for applied relaxation training with cognitive therapy on social phobia patients with fear of blushing, trembling, and sweating. Although both treatments were effective in reducing fear of body sensations, dysfunctional beliefs, self-consciousness, and self-focused attention, the effects for applied relaxation were smaller. Clark et al. (2006) found that cognitive therapy had greater effects in treating social phobia than a combination of exposure and applied relaxation therapy, as measured by the number of participants who met DSM criteria for social phobia after treatment and at followup. In a sample of individuals with hypertension, Achmon et al. (1989) compared CBT training to reduce anger with biofeedback and found a greater decreases in anger among individuals assigned to CBT training during exposure to stress. Effects of CBT as compared to psychophysiological interventions on medical conditions characterized by stress may also be greater. Deale et al. (2001) found greater five-year effects for CBT than progressive muscle relaxation therapy for reducing symptoms of chronic fatigue syndrome. Bracardi (1997) studied patients in a cardiac rehabilitation program. They found that psychoanalytically oriented group psychotherapy had a greater positive impact on motivation to engage in cardiac rehabilitation than frontal electromyographic biofeedback, with fewer dropouts both from the psychological treatment and from the entire cardiac rehabilitation program. Both approaches produced decreases in a measure of anxiety compared with a control group.

Results for psychophysiological therapies (primarily relaxation therapy) as monotreatment for panic show surprisingly weaker effects than cognitive and exposure therapies. Michelson et al. compared of the effects of relaxation therapy, exposure therapy and paradoxical intention on cognitive symptoms of panic. They found marginally greater cognitive effects following exposure and paradoxical intention interventions than for relaxation therapy (Michelson et al., 1988). However, when comparing psychophysiological improvements, greater effects were found for relaxation (Michelson et al., 1990). Barlow et al. (1989) found that cognitive therapy was more effective in reducing panic symptoms than relaxation training, although both treatments produced equivalent results for generalized anxiety symptoms. Arntz and van den Hout (1996) found a greater effect for cognitive therapy than relaxation therapy for treating panic disorder without agoraphobia. Beck et al. (1994) found a slightly greater effect for cognitive therapy than relaxation therapy for treating panic disorder with concurrent agoraphobic symptoms. Siev and Chambles (2007, 2008) found greater effects of exposure-based cognitive therapy than relaxation therapy for treating panic disorder. Carlbring et al. (2003) found marginally stronger effects on panic disorder for applied relaxation than for CBT, with a much larger effect size but nonsignificant between-group differences due to small sample size. Montero-Marin et al. (2018) found greater effects for CBT than relaxation therapy for PTSD, panic, and obsessive–compulsive disorder. Murphy et al., 1998) compared relaxation or cognitive therapy as added components to exposure therapy for treating panic disorder with agoraphobia. They found a slightly greater effect for cognitive therapy, significant only for some outcome measures.

However, none of these studies finding inferior effects for psychophysiological therapy on panic examined the effect of breathing therapies aimed at decreasing hyperventilation. This is a critical omission because of the large overlap between symptoms of panic and those of hyperventilation (Meuret & Ritz, 2010). Craske et al. (1997) found that exposure therapy involving deliberate hyperventilation was more effective than a combination of cognitive restructuring, breathing retraining, and exposure to feared situations. In a meta analysis of studies on breathing therapies for anxiety, Leyro et al. (2021) found that the one study doing biofeedback for decreasing end-tidal carbon dioxide had an outlyingly large effect size for treating panic.

Some studies found equivalent effects for psychophysiological and cognitive and/or behavioral interventions. Eisen and Silverman (1993) found equivalent effects for cognitive therapy and relaxation therapy for reducing anxiety among overanxious children. Ale et al. (2015) found in a meta-analysis that neither relaxation therapy nor CBT added significantly to the effects of exposure and response prevention interventions for childhood anxiety disorders, when added after the latter interventions. In a meta-analysis, Fluckiger et al. (2022) found no differences between CBT and relaxation therapy for treating generalized anxiety disorder at follow-up periods. In their meta-analysis, Montero-Marin et al. (2018) found no difference between relaxation therapy and CBT for generalized anxiety disorder, social anxiety disorder, and specific phobias. Beck et al. (1994) found no differences between relaxation therapy and cognitive therapy for treating panic disorder. Ost and Breitholtz (2000) found no differences between relaxation therapy and cognitive therapy for treating generalized anxiety disorder. Siev and Chambles (2007, 2008) found equivalent effects for cognitive therapy and relaxation therapy for treating generalized anxiety disorder. Ost and Westling (1995) found no differences between relaxation therapy and cognitive therapy for treating panic disorder. In a small study, Davies et al. (1995) found no differences between relaxation therapy and cognitive therapy for treating tinnitus. Carlbring et al. (2003) found no differences between internet-administered CBT and applied relaxation for treating panic disorder. Bell et al. (1983) compared three conditions for treating patients with tension headaches: broad based biofeedback, eclectic psychotherapy, and a combination of the two. They found equivalent effects on the amount of headache activity and various psychophysiological measures compared with a control group. Slavin-Spenny et al. (2013) found equivalent effects for relaxation therapy and training in anger awareness and expression for people with headaches, with improvement in both conditions. Several recent reviews (Banerjee & Argaez, 2019; Penzien & Irby, 2024) concluded that various psychotherapies and psychophysiological therapies, usually combined with pharmacotherapy, are all helpful in treating various pain conditions, but there are no general conclusions about advantages of one approach compared with another. In a meta-analysis, Guo et al. (2021) found that relaxation therapy, cognitive therapy, yoga, and mindfulness training all had significant but equivalent effects in reducing symptoms of stress in pregnant women. In a meta-analysis by Jia et al. (2020), effects of relaxation therapy on depression were found to be significant and equivalent to the effects of psychotherapy. Bowers (1990) studied depressed patients and compared nortriptiline alone with the medication plus cognitive therapy and medication plus relaxation therapy. Both treatment groups did better than the nortriptiline alone group, and the two behavioral treatments were equivalent for depressive symptoms, although the cognitive therapy group did better than the relaxation group in number of patients judged as depressed at Discharge. Murphy et al. (1995) found equivalent effects for CBT and relaxation therapy for treating depression, but both methods produced greater effects than treatment by tricyclic antidepressant drugs. In a meta-analysis of HRVB studies, Lehrer et al. (2020) found that heart rate variability biofeedback, overall, had equivalent effects to other effective treatments.

### Studies Adding Cognitive Behavioral Interventions to Psychophysiological Interventions

Olson and Malow (1987) compared electromyographic biofeedback with biofeedback plus cognitive behavioral stress management therapy for treating patients with myofascial pain dysfunction that did not respond to conventional physical treatment. They found that the addition of psychotherapy produced greater decreases in pain than biofeedback alone. Blanchard et al. (1990a) studied migraine headache patients, and compared remote thermal biofeedback, thermal biofeedback plus remote cognitive stress management instructions, and a group that just monitored headaches. Headache activity decreased in both treatment groups compared to the control group, with no differences between groups. In another study on a similar population (Blanchard et al., 1990b) compared progressive muscle relaxation instruction alone with progressive muscle relaxation plus cognitive therapy, compared with headache monitoring and a placebo condition. Both treatments showed greater decreases in headache activity than both control groups, with no differences between treatment groups. Mosley et al. (1995) found that adding CBT to relaxation therapy among patients with tension headaches yielded greater effects for headaches, depression, and minor stress, but equivalent effects for anxiety and anger. Attanasio et al. (1987) compared relaxation therapy, cognitive therapy, and the combination of the two for patients with tension headaches. They found improvements in all three conditions with no differences among the three. We found no more recent studies on additive effects of psychotherapies to relaxation methods.

## Discussion

With some differences found among studies and reviews, psychophysiological methods tend to be at least as helpful as cognitive behavioral or other psychotherapeutic for treating a variety of conditions, and seem to strengthen the effects of these other methods when combined with them. The effects appear stronger for anxiety and depression, and weaker for panic, PTSD, and perceived stress, particularly where treatments were mostly abbreviated forms of progressive muscle relaxation. The effects are weaker among children than among adolescents and adults. Adding biofeedback may have additional value for anxiety and depression, although effects also appear weaker in comparison to methods that include a component of exposure therapy. In general, however, the results support using a combination of psychophysiological measures with psychotherapy to obtain the strongest effects.

The surprisingly weaker results for panic than CBT may reflect which methods are studied and how they are used. Relaxation during the height of a panic attack may have trivial effect, since hyperventilation is a core mechanism of panic symptoms (Sikter et al., 2007), and it may worsen if metabolic rate is decreased by relaxation without a compensatory decrease in ventilation. Transitory hyperventilation often does occur early in training for respiratory interventions, as an overcompensation for slower breathing. Although standard biofeedback protocols call for training to breathe shallowly during slowed breathing (Lehrer et al., 2013), this treatment component is often omitted. Patients with panic symptoms may have a greater tendency to overbreathe, thus compounding this hyperventilation effect. Respiratory interventions, particularly biofeedback to increase end-tidal carbon dioxide and heart rate variability biofeedback, appear to have particularly strong effects (Leyro et al., 2021; Meuret et al., 2008), although the effects have not been systematically studied during panic attacks, and the results have not been compared with other forms of therapy.

Variability among findings may be due to a variety of factors, including length and intensiveness of training. Few studies used the more intensive methods of psychophysiological treatment described at the outset of this paper. Also, there is considerable variability of effects *within* studies, probably reflecting differences among individuals in how they relate to any of the therapies studied, as well as differences in ways that outcome was assessed.

Psychophysiological interventions as monotherapies may not be the most effective approach. Adding other forms of psychotherapy and/or pharmacotherapy to psychophysiological methods appears to strengthen the effects of therapy, just as adding psychophysiological therapy to other forms of psychotherapy enhances effects of these other methods. Combined use of therapy modalities appears to have the strongest effects.

Further research is needed to confirm these findings. Studies comparing and/or adding respiratory therapies to psychotherapy are lacking, as are studies focusing on nonpharmacological modification of other core physiological problems for various populations and disorders. Particularly important would be studies that include biofeedback to increase exhaled carbon dioxide and/or heart rate variability. Additionally, more studies are needed comparing the more rigorous treatment methods described in this paper to the more approximate methods in widespread use, as was done in two studies on HRVB but none for progressive muscle relaxation or autogenic training. This would confirm whether the more rigorous method has measurable benefits, as it apparently does for HRVB.

## Data Availability

No datasets were generated or analysed during the current study.
